# Effect of Nutritional Habits on the Glycemic Response to Different Carbohydrate Diet in Children with Type 1 Diabetes Mellitus

**DOI:** 10.3390/nu13113815

**Published:** 2021-10-27

**Authors:** Agnieszka Lejk, Jędrzej Chrzanowski, Adrianna Cieślak, Wojciech Fendler, Małgorzata Myśliwiec

**Affiliations:** 1 Department of Paediatrics, Diabetology and Endocrinology, Medical University of Gdańsk, 80-210 Gdańsk, Poland; 2 Department of Biostatistics and Translational Medicine, Medical University of Lodz, 92-215 Lodz, Poland; jj.chrzanowski1@gmail.com (J.C.); adrianna.m.cieslak@gmail.com (A.C.); wojciech.fendler@umed.lodz.pl (W.F.)

**Keywords:** paediatric diabetes, carbohydrates, individualized nutritional guideline

## Abstract

Unhealthy eating habits are associated with obesity, metabolic syndrome, and increased insulin resistance in young patients with type 1 diabetes mellitus (T1DM), and may impact the possible benefit from dietary interventions on glycaemic control. This study determines how nutritional patterns influence the quality of dietary intervention with a 30% or 50% carbohydrate diet in terms of glycaemic control measured with continuous glucose monitoring (CGM). Eating habits were obtained with a frequency-of-consumption questionnaire (FFQ-6) before the diet assessment. Altogether, we collected CGM and FFQ-6 data from 30 children (16 boys and 14 girls aged 10–17 years) with T1DM subjected to two consecutive 3-day nutritional plans. From these, 23 patients met the CGM data quality criteria for further analysis. Furthermore, high accuracy achieved in training (95.65%) and V-fold cross-validation (81.67%) suggest a significant impact of food habits in response to introduced nutritional changes. Patients who consumed more vegetables or grains (>4 times per day), more wheat products (>once per day), fewer fats (<1.5 times per day), and ranked fruit juice as the most common selection in the drinks category achieved glycaemic control more often after the introduction of a 30% carbohydrate diet, as opposed to those with different dietary patterns, whose glycaemic control was negatively impacted after switching to this diet. Additionally, the 50% carbohydrate diet was safe for all patients in the context of glycaemic control.

## 1. Introduction

Diabetes mellitus type 1 (T1DM) is an autoimmune disease in which pancreatic beta cell destruction leads to an absolute insulin deficiency, resulting in dysregulation of glycaemic control [1]. If left poorly controlled, it may cause life-threatening events, such as diabetic ketoacidosis and nonketotic hyperosmolar coma, with long-term complications including heart disease, neuropathy, nephropathy, retinopathy, or even death [2]. T1DM is one of most common noninfectious chronic diseases among Polish children [3]. In the last five years, the incidence of T1DM has increased 1.5-fold in the population under 18 years old in Poland [4].

Currently, there are no effective or clinically useful methods that could prevent T1DM, both in the general population and in subjects at risk. The only treatment is insulin replacement therapy with either multiple daily injections (MDI) or continuous subcutaneous insulin infusion (CSII) using a personal insulin pump [5]. According to Diabetes Poland, children and adolescents with T1DM should be treated with intensive insulin therapy, and use continuous glucose monitoring (CGM) systems from the onset of the disease to improve the metabolic control of diabetes, and decrease the risk of acute and chronic complications [6].

In addition to insulin therapy, proper nutrition and exercise play a significant role in diabetes treatment [7]. Dietary recommendations are similar to the principles of proper nutrition for healthy children. Nevertheless, Patton’s studies [8] examined macronutrients and dietary recommendations in children with T1DM, which revealed higher than the recommended intakes of fat, and lower than the recommended intakes of fruits, vegetables, and whole grains. Nansel et al. [9], and Meissner et al. [10] reached similar conclusions.

According to the Clinical Recommendations of Diabetes Poland 2021, carbohydrates should cover 45–50% of the daily energy requirement, and the simple sugars contained in them should not exceed 10%. Proteins should cover 15–20% of daily caloric intake, and fats should cover 30–35% [11]. Numerous recent studies have emphasised the role of a properly balanced diet with a slight reduction in carbohydrate intake, especially simple sugars, as a part of the treatment of T1DM. Krebs et al. [12], and Seckold et al. [13] suggested that reducing carbohydrates in the diet may benefit glycaemic control.

Nutritional challenges in the Polish paediatric population have not changed much since the COVID-19 pandemic. Inappropriate food habits, which include the consumption of fruit juices, carbonated beverages, diet sodas, fast food, snacks, and sweets, still remain a major problem. Moreover, a sedentary lifestyle during the pandemic has predominated over physical activity and has led to obesity among Polish girls and boys (14% and almost 20%, respectively) [14]. One of the studies conducted between 2008 and 2016 in the municipality of Gdańsk showed that the prevalence of overweight and obesity among 6- to 7-year-old children was 7.49 and 4.24%, respectively [15]. There is a lack of educational and interventional programmes in Poland concerning the prevalence of excess body weight and its consequences in children. One of few such intervention programmes is “6-10-14 for Health” for obese children from the Gdańsk municipality, where both participants and their family members are offered a 12-month integrated intervention, including medical, dietetic, and psychological counselling, and educational workshops for parents [16].

Some studies conducted during the pandemic showed quite a different perspective. Głąbska et al. concluded that the COVID-19 era may have changed the determinants of food choices in Polish adolescents, as it may have increased the importance of health and weight control [17]. Łuszczki et al. had a similar opinion and even emphasised that dietary patterns are better now than they were before the pandemic [18]. Although these studies give hope for improving the overall nutritional approach among Polish children, the problem of inappropriate eating patterns is still very common.

This study determines how nutritional habits influence glycaemic control during the dietary intervention introduction of a 30% or 50% carbohydrate diet with maintained total caloric intake, using the continuous glucose monitoring system.

## 2. Materials and Methods

### 2.1. Participants, Recruitment, and Study Design

We recruited paediatric patients, median age 16 (13–17 years), with diagnosed T1DM according to the criteria of International Society for Pediatric and Adolescent Diabetes (ISPAD) guidelines [19] with diabetes duration of at least one year and HbA1c level ≤9.0% (75 mmol/mol), remaining under the care of the Clinic of Paediatrics, Diabetology, and Endocrinology at the Medical University of Gdańsk, Poland.

Exclusion criteria for participation in the study were concomitant chronic diseases associated with hypoglycaemia or special dietary requirements (e.g., hypothyroidism, liver, renal disorders, and coeliac disease).

Two consecutive 3-day nutritional plans were created for each patient, with carbohydrates covering 30% or 50% of the daily energy requirement. Diets were composed and evaluated using diet programme Aliant, and scored using the menu score. Each patient participated in both nutritional interventions, one after another (cross-over and cross-sectional design), with no wash-out period in between.

The Aliant programme is a professional diet calculator for dieticians and nutrition specialists who plan and evaluate individual nutrition. The manufacturer created their own product database (3700 items: products and ready meals), which is constantly expanding, and introduced meal units in the form of patient-friendly home measures; currently, there are 3200 of them for over 1900 products. The diet can be implemented for up to 30 days with a maximum of 10 meals a day, with the possibility of setting a different number of meals each day [20].

Both diets were created in accordance with the nutritional standards for Polish children, taking into account the patient’s gender, age, and physical activity [21]. In planning the diets, attention was paid to the principles of a diet with a low glycaemic index. Each patient would eat a 30% carbohydrate diet during the 3-day hospitalisation and a 50% carbohydrate diet at home.

**Figure 1:**
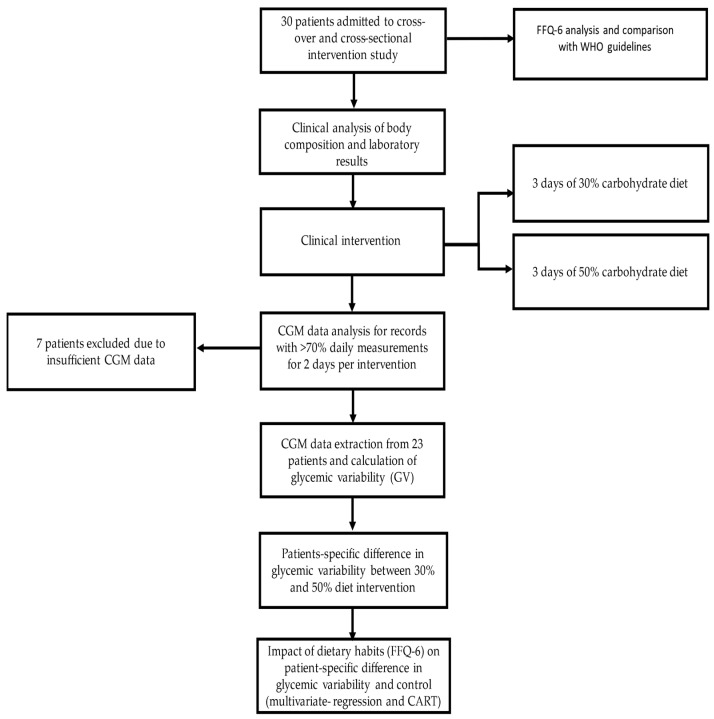
Study design and analysis diagram.

Patients were carefully monitored during the dietary interventions by a dietitian, diabetologist, nurse, and the principal investigator. Subjects were required to strictly follow the nutrition plan and avoid any additional food consumption. During the experiment, their blood glucose was measured using the continuous glucose monitoring (CGM) system and the self-monitoring of blood glucose (SMBG). The CGM measured real-time interstitial glucose concentration and was not blinded, so the diabetologist and the patients had continuous access to glucose concentrations. The diabetologist and dietician calculated the daily insulin intake (insulin base and boluses) for each patient to provide optimal glycaemic control. Adverse events, defined as severe hypoglycaemia, diabetic ketoacidosis, or allergic reactions, were registered for all patients during and 48 h before and after the dietary intervention. Detailed scheme of the study is presented in Figure 1.

### 2.2. Ethics Statement

The study protocol was approved by the Bioethical Commission of the Medical University of Gdańsk (no. NKBBN/299/2019). Each participant and their parents were informed of the study protocol by the principal investigator and signed the written consent form.

### 2.3. Collecting Clinical and Food Preferences Data

Dietary habits were assessed by the frequency-of-consumption questionnaire (FFQ-6), which was obtained from patients under the close supervision of the principal investigator. It is generally used to collect information on the frequency of consumption of 62 assorted product groups, representing 8 major food groups (sweets and snacks, dairy products and eggs, grains, fats, fruits and vegetables, meat and fish, beverages). Respondents chose one of six potential answers regarding their food consumption frequency in the last 12 months: (1) never or hardly ever, (2) once a month or less frequently, (3) several times a month, (4) several times a week, (5) daily, (6) several times a day [22]. Obtained data were in rank format; therefore, we recoded them into appropriate frequencies, which were illustrated in the description of the characteristics of the questionnaire. Calculated frequencies from the questionnaire were summed up within the categories, adjusted into portions per day, and compared with the European recommendations.

Patients underwent body composition analyses on enrolment using a TANITA SC-240 MA body composition analyser and the complete clinical evaluation by the paediatric diabetologist at the study initiation and completion. Additionally, before the experiment, blood samples were taken from each patient within standard clinical assessment procedures. Lipid levels, glycated haemoglobin (HbA1c), vitamin D (25-OHD), and liver enzymes (ALAT, AST) from each patient were assayed. HbA1c was assessed via high-performance liquid chromatography (HPLC) with traceable agreement to the Diabetes Control and Complications Trial according to the NGSP program (D-10 Haemoglobin A1c Program; Bio-Rad Laboratories, Hercules, CA, Bio-Rad, Marnes-la-Coquette, France). The concentration of 25-OHD was assessed by a two-step immunochemical method using microparticles and CMIA chemiluminescent tracer (Abbott Laboratories, Germany). Liver enzymes (ALAT, AST) were assessed via HPLC.

### 2.4. Collecting Continuous Glucose Monitoring Data

CGM systems allow for the more effective adjustment of insulin doses, which provides information to administer the best possible glycaemic control for the patient, especially in a hospital setting [23]. We utilised CGM sensors (Enlite real-time glucose sensor) to measure glycaemic variability proxy: daily mean glucose, standard deviation (SD), coefficient of variation (CV), and time in range (TIR) [24]. Moreover, all patients in the study were treated with insulin pumps (Medtronic Paradigm 722, Paradigm Veo 754 or MiniMed 640G), which are standard for diabetes care in Poland, where over 70% of paediatric type 1 diabetes patients are treated with pumps, one-third of which use pump integration with CGM sensors [25,26].

Patients’ CGM data were included in the analysis only if their CGM records satisfied the quality criteria, defined as at least 70% of daily sensor active time for at least two days of each dietary intervention. Glycaemic variability was computed using the authors’ implementation of glycaemic variability indices, as defined in the American Diabetes Association’s guidelines [1,6].

### 2.5. Statistical Analysis

Continuous variables are presented as means ± standard deviations or median with first and third quantiles, and tested using a t-test or Mann–Whitney’s U test depending on normality. Nominal variables were tested using Fisher’s exact test.

Changes in glycaemic variability were tested using mixed linear models with a diet (30%, 50%) as a fixed effect and the patient ID as a random effect. In addition, correlation between continuous variables was tested using Pearson r-statistics.

The impact of dietary habits on the difference between glycaemic variability in the dietary intervention was defined as:

ΔGV = GV(diet 50%) − GV(diet 30%)(1)

The impact of dietary habits on glycaemic control was also evaluated using classification and regression trees (CART) analysis, which allows for the automated supervised determination of variables providing the best discrimination of the binary outcome. The benefit of CART over logistic regression is the possibility to investigate nonlinear interactions between variables and outcomes. The application of CART allowed for the best separation of patients with gain in glycaemic control in a specific dietary intervention based on their assessed nutritional habits. Glycaemic control is defined here as time in range 70–180 mg/dL > 70% and CV% < 36%. Results from CART are provided using decision-tree visualisation. As this is a proof-of-concept study, we had no external cohort for independent validation. As such, we applied V-fold cross-validation. Error metrics were reported according to the TRIPOD checklist.

All statistical analyses were performed in Python 3.8 and STATISTICA 13.1 (Dell Inc., Round Rock, TX, USA). The *p* value was considered to be significant at <0.05. No correction for multiple comparisons was applied.

## 3. Results

### 3.1. Study Group Characteristics

A total of 30 patients (16 boys and 14 girls) were enrolled in the study, with a median age of 16 (13–17 years). All patients were in the pubertal period, with the majority in the fourth (*n* = 8, 26.67%) and fifth (*n* = 16, 53.33%) Tanner classes. Most of the patients (26, 86.67%) were within 95 centiles for BMI for age and sex; 1 patient was underweight (BMI centile = 8.15), and 3 were obese (BMI centile = 97.069; 96.130; 97.683). All patients were Caucasian, and on clinical evaluation reported only type 1 diabetes, with no significant concomitant diseases or signs of malnutrition. No adverse events were observed in the week before, during, and the week after the introduction of dietary plans. The detailed clinical characteristics of the group are presented in Table 1.

### 3.2. FFQ-6 Results and Interpretation

The dietary patterns of the patients collected from the FFQ-6 are shown in Table 2. It appears that children with T1DM had difficulty following European recommendations on the appropriate amount of macronutrients in their diet. The least number of patients followed the EU recommendations in the context of bread, grains and potatoes consumption (6.67%), whereas 70% followed the restrictions on meat and fish daily intake. Another alarming aspect is the high consumption of sweets and snacks, which are classified as high carbohydrate foods.

### 3.3. Glycaemic Variability Analysis Based on Used Diet

**Figure 2:**
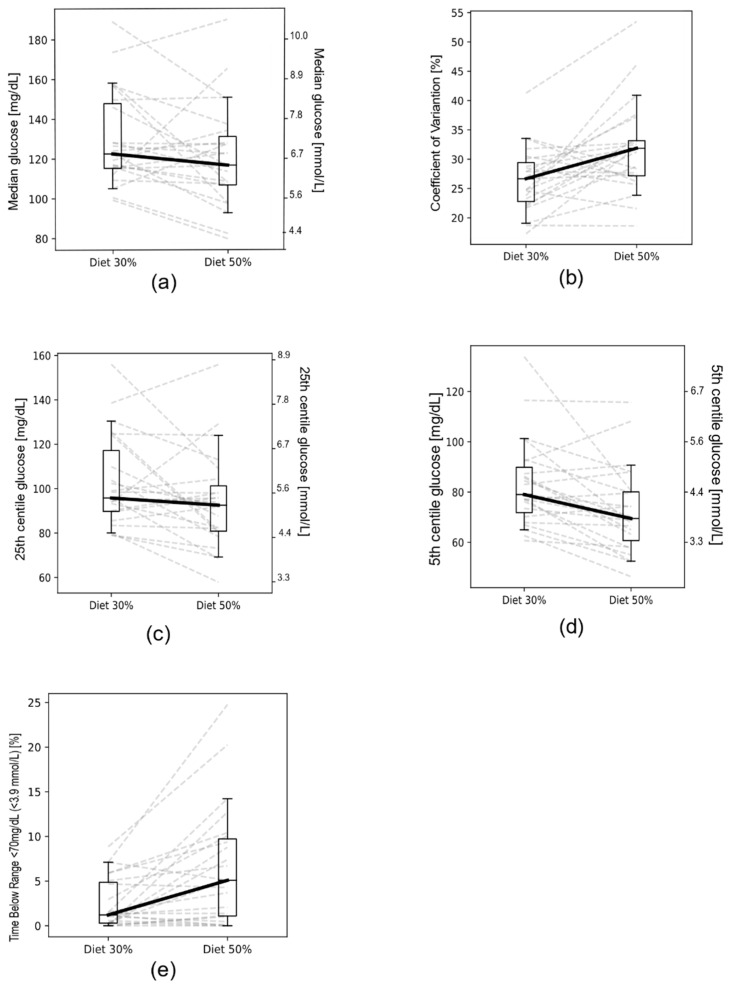
Comparison between particular glycaemic variables for 30% and 50% diets: (**a**) median; (**b**) coefficient of variation (%); (**c**) 25th cent.; (**d**) 5th cent.; (**e**) time below range 70 mg/dL (<3.9 mmol/L). Dashed lines represent parameter changes between diets, as specified per individual patients.

After the initial check for CGM record quality, only 23 patients were eligible for a comprehensive assessment of glycaemic variability between dietary plans, giving 111 observations (24 h CGM) in total, as presented in Table 3. Briefly, significant differences were found for: median, CV%, 5th and 25th centiles of glucose, and time below target range (<70 mg/dL; <3.9 mmol/L). A comparison of glycaemic variability indices between diets is shown in Figure 2.

### 3.4. Analysis of Glycaemic Variability Indices between Diets in the Context of Nutritional Habits

Of the 30 children with T1DM included in the study, only 23 participants met the CGM data quality requirements, providing a total of 111 observation days. To discern differences observed in glycaemic variability between the diets, we utilised data on nutritional habits from the FFQ-6 questionnaire. The high consumption of fruit juices and carbonated drinks decreased the risk of hypoglycaemia in the 30% carbohydrate diet, as measured by the percentage of time spent below 70 mg/dL (<3.9 mmol/L) (*p* = 0.0401, 0.0183, respectively) and the 5th centile of daily glucose values (*p* = 0.0209, 0.0401, respectively). The full results of the nominal variable analysis are provided in Supplementary Table S1.

### 3.5. Individual Response to Low Carbohydrate Diet Predicted by FFQ-6 Questionnaires

**Figure 3:**
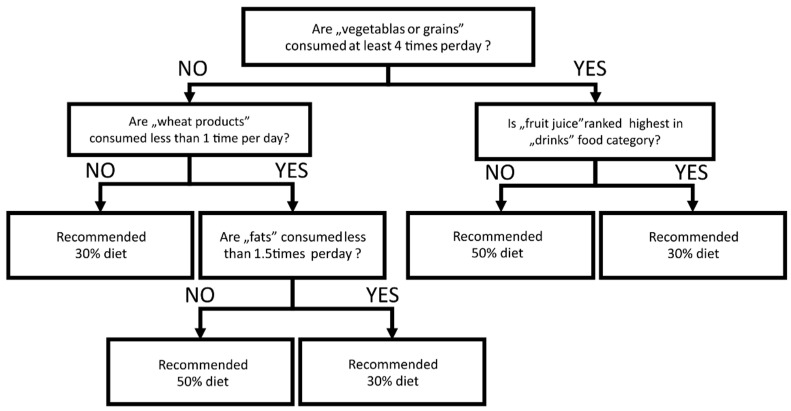
Decision-tree diagram for classification and regression trees (CART) analysis. Influence of FFQ-6 measured nutritional habits in the benefit of glycaemic control after dietary intervention (30% or 50% carbohydrate diet).

To determine which patient would benefit the most from a 30% carbohydrate diet, we applied the CART model to the binary outcome of the glycaemic control (defined as CV < 36% and TIR70-180 mg/dL > 70%). CART analysis identified that patients consuming more vegetables or grains (>4 times per day), more wheat products (>once per day), fewer fats (<1.5 times per day), and ranking fruit juice as the most common selection in the drinks category, achieved glycaemic control more often after the introduction of the 30% carbohydrate diet (Figure 3). In contrast, if patients were not conditioned in the described way, the switch to the 30% carbohydrate diet could negatively impact glycaemic control. In comparison, the 50% carbohydrate diet was safe for all patients in the context of glycaemic control.

The CART model yielded satisfactory accuracy on training (95.65%) and V-fold cross-validation (81.67%). We also aimed to discern which nutritional and clinical information could determine the difference between the 30% and 50% diets (ΔGV, as described in Equation (1)). The backward stepwise feature selection algorithm determined included features in each multifactor linear regression for the selected ΔGV indices (mean glucose, coefficient of variation (%), time below target range <70 mg/dL (<3.9 mmol/L), 70–180 mg/dL (3.9–10 mmol/L) and >180 mg/dL (>10 mmol/L)). Each model was validated with V-fold cross-validation. We summarised model errors with the determination coefficient R^2^ corrected for the number of features (Table 4).

## 4. Discussion

These diets were prepared following the principles of a low glycaemic diet considering starch resistance and fixed mealtimes [10] Insulin doses were administered to children during the carbohydrate portions to avoid any combined or extended boluses. Additionally, patients drank at least 2 litres of still water per day. All patients had the same physical activity during the experiment to exclude additional factors affecting glucose variability. We registered no acute hypo- or hyperglycaemic events during the study.

According to the frequency of FFQ-6 data, participants had similar eating habits and made comparable nutrition mistakes when it came to the consumption of carbohydrates in comparison with the amount of protein and fat in their diets.

Our study focused on the impact of glycaemic variability while introducing children with T1DM to specific diets using a continuous glucose monitoring system. There is still no recommendation on how to safely introduce a low-carbohydrate diet to children with T1DM. Therefore, we investigated this issue in a small group of children with T1DM.

Significant differences in CV% and TBR (<70 mg/dL; <3.9 mmol/L) between the two diets suggested that there is potential for better glycaemic control on low-carbohydrate (30%) intake. Souza Bosco Paiva et al., and Lennerz et al. [27,28] came to a similar conclusion. Furthermore, they observed better HbA1c and glycaemic control in children with T1DM on a very low carbohydrate diet [21]. In contrast, other authors paid more attention to an individual approach to the patients’ and their bodies’ demand for carbohydrates [29,30].

Median, 5th, and 25th centile glucose values were better for the 50% carbohydrate diet (though the difference was not statistically significant). Moreover, interpatient variability in the ΔGV is of relevant magnitude to consider possible unaccounted biases. One such source of bias might be the difference in the dynamics of energy consumption or errors in BMR estimation (BMR was based on TANITA’s algorithm). Both factors could be conditioned by physical fitness (i.e., differences in muscle mass or muscle-to-fat ratio), which was not accounted for in the data collection protocol. The role of activity in maintaining metabolic control was demonstrated by Myśliwiec et al. [31]. Their research showed that physical activity is a critical factor in controlling glycaemic excursions in young males with T1DM. The same results were emphasised in many in several previous publications, for example, Riddell et al. [32], and Bally et al. [33].

However, both the quantity and quality of carbohydrates are of paramount importance. Patients with a previous high consumption of fruit juices and carbonated drinks had a lower risk of time spent below the optimal glucose level range (TIR) (*p* = 0.0401, *p* = 0.0183, respectively), and their 5th centile of daily glucose values was higher in the 30% carbohydrate diet (*p* = 0.0209, *p* = 0.0401, respectively). The same phenomenon describing this tendency was observed by de Bock et al. [34], and Mansoor et al. [35].

Furthermore, our study emphasised the importance of properly balanced meals. The choice of carbohydrates and how they are prepared are keys to achieving better glycaemic control. Reynold et al. [36], Sterner Isaksson et al. [37], and Nansel et al. [9] had the same suggestions. They observed that, with adequate intake of dietary fibre, low-glycaemic-index products resulted in a lower tendency of hypo- and hyperglycaemia.

Moreover, using CART analysis and FFQ-6, we could determine which patients may benefit the most from going on a 30% diet, or staying or aiming for a 50% diet. Patients consuming more vegetables or grains (>4 times per day), more wheat products (>once per day), fewer fats (<1.5 times per day) and drinking fruit juice as the most common beverage achieved glycaemic control more often after the introduction of a 30% carbohydrate diet. High accuracy achieved in training (95.65%) and V-fold cross-validation (81.67%) suggested a significant impact of food habits on patients’ response to introduced nutritional changes. Our observation showed that a well-balanced diet under the principles of proper nutrition helps clinicians control glycaemic variability among patients with T1DM and prevents the occurrence of hypoglycaemia. This beneficial role of the average amount of carbohydrates is explained well in other studies [38,39].

## 5. Conclusions

Patients consuming fewer carbohydrates are better metabolically balanced. Thus, despite the limited observation time for introduced diets, our research confirmed that proper nutrition and an individual approach to a patient’s diet results in better metabolic control. However, given that this is the first study suggesting that eating patterns may influence the adjustment to a low-carbohydrate diet, further research is required before developing definitive recommendations around an optimal nutritional programme for children with T1DM.

## Figures and Tables

**Table 1:** Study group characteristics.

Category (*n* = 30)	Median (25–75%)	Min–Max
Age [years]	16.00 (13.00–17.00)	10–17
Disease time [years]	6.00 (3.00–8.00)	1.00–15.00
BMI centile	78.21 (55.62–89.94)	8.15–97.68
Time using pump [years]	3.00 (1.00–7.00)	1.00–14.00
Initial HbA1c [%]	7.25 (6.90–7.70)	5.40–8.10
Initial HbA1c [mmol/mol]	55.738 (51.913–60.656)	35.519–65.027
Mean daily insulin requirement [u/day/kg of weight]	0.75 (0.59–0.90)	0.20–1.40
Body fat % [Tanita]	20.30 (14.50–29.20)	12.70–39.00
AST [U/L]	17.00 (15.00–19.00)	12.00–40.00
ALAT [U/L]	13.00 (10.00–15.00)	5.00–26.00
TC	169.0 (149.0–188.0)	119.0–257.0
LDL	94.50 (75.0–107.0)	44.0–175.0
HDL	61.50 (52.0–70.0)	30.0–92.0
TG	71.0 (62.0–90.0)	28.0–181.0
Vitamin D ng/mL	21.65 (18.00–27.80)	6.00–40.70

BMI centiles were determined sing an appropriate calculator.

**Table 2:** Characteristics of the Food Frequency Questionnaire 6 (FFQ-6) responses according to WHO food-based dietary guidelines and WHO sugar intake for adults and children guidelines.

FFQ-6 Category	Median (25–75 Cent.) Portions/d	Min–Max Portions/d	European Recommendations Portions/d	N (%) below the EU Recommendations	N (%) of Patients that Achieved EU Recommendations	N (%) above the EU Recommendations
Meat and fish	1.6 (0.7–2.2)	0.1–3.1	1–2	4 (13.33%)	21 (70.00%)	5 (16.67%)
Fats	2.2 (1.7–2.7)	0.4–5.2	2	5 (16.67%)	12 (40.00%)	13 (43.33%)
Fruits	2.6 (1.7–3.5)	0.1–8.3	~1–2	3 (10.00%)	11 (36.67%)	16 (53.33%)
Fruits and vegetables	5.4 (3.6–6.8)	0.1–15.6	~5–6	7 (23.33%)	11 (36.67%)	12 (40.00%)
Dairy products and eggs	2.3 (1.4–2.8)	0.8–5.7	3–4	18 (60.00%)	9 (30.00%)	3 (10.00%)
Sweets and snacks	0.9 (0.6–1.7)	0.2–3.5	0	-	5 (16.67%)	25 (83.33%)
Vegetables	2.5 (1.5–3.3)	0.0–10.0	~4–5	23 (76.67%)	4 (13.33%)	3 (10.00%)
Bread, grains, potatoes	3.1 (2.3–3.6)	2.0–5.1	~5–6	28 (93.33%)	2 (6.67%)	-

**Table 3:** Comparison of glycaemic parameters based on used diet.

GV	Diet 30% Mean ± SD (*n* = 23)	Diet 50% Mean ± SD (*n* = 23)	Change for Diet 30% Mean ± SD (*n* = 111)	*p*
Mean glucose (mg/dL)	133.16 ± 23.94	125.95 ± 22.88	+5.77 ± 22.70	0.0620
Median glucose (mg/dL)	129.78 ± 23.46	120.95 ± 25.49	+6.17 ± 21.53	0.0470
25th centile glucose (mg/dL)	105.14 ± 20.24	96.00 ± 21.49	+5.23 ± 16.81	0.0060
75th centile glucose (mg/dL)	156.75 ± 29.09	149.02 ± 26.64	+5.69 ± 31.72	0.1450
5th centile glucose (mg/dL)	83.40 ± 17.11	72.69 ± 16.92	+6.69 ± 16.30	0.0001
95th centile glucose (mg/dL)	197.52 ± 39.39	201.43 ± 36.91	+9.37 ± 57.84	0.6280
SD (mg/dL)	35.4 ± 9.653	39.39 ± 10.12	+0.16 ± 16.38	0.0750
CV (%)	26.50 ± 5.43	31.68 ± 7.57	−2.45 ± 8.12	0.0005
Time below target range <54 mg/dL (<3 mmol/L) (%)	0.43 ± 0.91	1.98 ± 3.91	+0.07 ±3.51	0.0950
Time below target range <70 mg/dL (<3.9 mmol/L) (%)	2.49 ± 2.76	6.49 ± 6.56	−1.01 ± 4.80	0.0003
Time in target range 70–180 mg/dL (%)	82.45 ± 13.83	77.89 ± 13.76	−1.65 ± 13.42	0.1440
Time in target range 180–250 mg/dl (>10 mmol/l) (%)	12.74 ± 11.93	11.88 ± 11.63	+1.58 ± 11.66	0.7460
Time above target range >250 mg/dl (>13.9 mmol/l) (%)	1.90 ± 3.22	1.77 ± 2.66	+1.02 ± 5.30	0.8270

Abbreviations: standard deviation (SD); coefficient of variation (CV); time above range (TAR); time below range (TBR); time in range (TIR).

**Table 4:** Coefficient determination (R^2^) and beta coefficients for multivariate linear regression models for change in selected glycaemic variability indices between the diets (ΔGVs).

ΔGV (50–30% Diet)	R2	Observed FFQ6 and Clinical Data Influence on ΔGV
Mean	0.5084	−0.40 × (white bread most common (0/1)) 0.57 × (frequency of meat/fish meals consumed per day)
CV%	0.4401	0.60 × (frequency of grains meals consumed per day) −0.42 × (frequency of drinks consumed per day)
TBR < 70 mg/dL	0.2453	0.53 × (frequency of grains consumed per day)
TIR 70–180 mg/dL	0.6398	0.42 × (white bread most common (0/1)) 0.35 × (potatoes most common (0/1)) −0.54 × (frequency of meat/fish meals consumed per day)
TAR > 180 mg/dL	0.6180	0.57 × (frequency of meat/fish meals consumed per day) 0.39 × (frequency of sweets and snacks consumed per day) 0.37 × (level of 25OHD3)

## Data Availability

Data presented in this study are available on request from the corresponding author. The data are not publicly available due to subjects’ privacy.

## Supplementary Materials

The following are available online at https://www.mdpi.com/article/10.3390/nu13113815/s1, Table S1: Summary and nominal *p*-values for FFQ-6 questionnaire responses.
