# Supplementary material for: Effect of Nutritional Habits on the Glycemic Response to Different Carbohydrate Diet in Children with Type 1 Diabetes Mellitus
Source: Nutrients. 2021 Oct 27;13(11):3815. doi: 10.3390/nu13113815 (PMC8620061; doi:10.3390/nu13113815)
Supplement: Supplementary file 1 [file nutrients-13-03815-s001.zip › nutrients-1375828-supplementary.pdf]

nutrients MDPI

Supplementary Table S1. Comparison of the most frequently consumed foods by patients in different diets with respect to glycemic parameters.

Category	The most frequently selected product	Median for 30% N = 15 (%)	Median for 50% N= 8 (%)	p-value	25cent for 30% N=16 (%)	25cent for 50% N=7 (%)	p-value	5cent for 30% N=18 (%)	5cent for 50% N= 16 (%)	p-value	CV (%) for 30% N=7 (%)	CV (%) for 50% N= 16 (%)	p-value	TBR70 for 30% N=6 (%)	TBR70 for 50% N=17 (%)	p-value
Sweets and snacks	Chocolate	10 (66.67%)	4 (50.00%)	0.657	11 (68.75%)	3 (42.86%)	0.363	11 (61.11%)	3 (60.00%)	1.00	4 (57.14%)	10 (62.50%)	1.00	3 (50.00%)	11 (64.71%)	0.643
Dairy products and eggs	Milk	8 (53.33%)	5 (62.50%)	1.00	8 (50.00%)	5 (71.43%)	0.405	11 (61.11%)	2 (40.00%)	0.6175	5 (71.43%)	8 (50.00%)	0.405	3 (50.00%)	10 (58.82%)	1.00
Grains	Whole meal bread	11 (73.33%)	4 (50.00%)	0.3712	12 (75.00%)	3 (42.86%)	0.1819	12 (66.67%)	3 (60.00%)	1.00	3 (42.86%)	12 (75.00%)	0.1819	4 (66.67%)	11 (64.71%)	1.00
	White bread	7 (46.67%)	7 (87.50%)	0.0858	8 (50.00%)	6 (85.71%)	0.176	10 (55.56%)	4 (80.00%)	0.6106	4 (57.14%)	10 (62.50%)	1.00	4 (66.67%)	10 (58.82%)	1.00
Fats	Butter	10 (66.67)	4 (50.00%)	0.657	11 (68.75%)	3 (42.86%)	0.363	11 (61.11%)	3 (60.00%)	1.00	4 (57.14%)	10 (62.50%)	1.00	4 (66.67%)	10 (58.82%)	1.00
Fruits	Apples	11 (73.33%)	7 (87.50%)	0.6214	12 (75.00%)	6 (85.71%)	1.00	13 (72.22%)	5 (100.00%)	0.5453	5 (71.43%)	13 (81.25%)	0.6214	5 (83.33%)	13 (76.47%)	1.00
Vegetables	Tomatoes	8 (53.33%)	5 (62.50%)	1.00	8 (50.00%)	5 (71.43%)	0.405	11 (61.11%)	2 (40.00%)	0.6175	6 (85.71%)	7 (43.75%)	0.0886	3 (50.00%)	10 (58.82%)	0.155
	Potatoes	8 (53.33%)	3 (37.50%)	0.6668	8 (50.00%)	3 (42.86%)	1.00	9 (50.00%)	2 (40.00%)	1.00	2 (28.57%)	9 (56.25%)	0.3707	1 (16.67%)	10 (58.82%)	1.00
Meat and Fish	Poultry meat	9 (60.00%)	4 (50.00%)	0.685	9 (56.25%)	4 (57.14%)	1.00	12 (66.67%)	1 (20.00%)	0.1269	4 (57.14%)	9 (56.25%)	1.00	3 (50.00%)	10 (58.82%)	1.00
Beverages	Fruit juices	14 (93.33%)	5 (62.50%)	0.1028	14 (87.50%)	5 (71.43%)	0.5573	**17 (94.44%)**	**2 (40.00%)**	**0.0209**	6 (85.71%)	13 (81.25%)	1.00	**3 (50.00%)**	**16 (94.12%)**	**0.0401**
	Carbonated drinks	5 (33.33%)	4 (50.00%)	0.657	6 (37.50%)	3 (42.86%)	1.00	6 (33.33)	3 (60.00%)	0.3428	3 (42.86%)	6 (37.50%)	0.3428	**5 (83.33%)**	**4 (23.53%)**	**0.0183**

[1] Fisher exact test was performed to obtain p-values. Statistically significant data was bolded ($p < 0.05$).
